# There may be a differential mechanistic impact on colorectal cancer of lactose-containing foods between lactase persistent and lactase non-persistent populations

**DOI:** 10.3389/fnut.2026.1671166

**Published:** 2026-02-11

**Authors:** Andrew Szilagyi, Polymnia Galiatsatos, Noah Margolese

**Affiliations:** 1 Division of Gastroenterology, Department of Medicine, Jewish General Hospital, McGill University, Montreal, QC, Canada; 2 Faculty of Medicine and Health Sciences, University of Sherbrooke, Sherbrooke, QC, Canada

**Keywords:** cancer, colon, digestion, lactose, milk

## Abstract

It is generally suggested that milk and milk products reduce the risk of colorectal cancer (CRC). While there is some controversy over specific sites affected throughout the colon and the benefits of specific dairy foods (DFs), there is a general consensus that calcium intake is the main mechanism of the cancer-reducing effects. This opinion may be sidelined by several other mechanisms. There is also a potentially important compensatory mechanism in populations with adult genetic lactase deficiency. The microbiome changes occur through a process of adaptation to continued lactose consumption. The bacterial blooms consist largely of *Bifidobacterial* species. These bacteria may exert anti-neoplastic effects and also increase the capacity of persons with adapted lactase insufficiency to consume dairy products. Bacterial metabolism thus provides a second pathway for lactose digestion. Since the use of Mendelian randomization (MR) accuracy disallow two different pathways for the genetic variable, this process constitutes a horizontal pleiotropy. This narrative review using articles from PubMed and Google Scholar will discuss different nutrients and mechanisms in milk and milk products that are involved in anti-neoplastic effects. The impact of adult lactase deficiency and continued dairy consumption on the microbiome, and its contribution to colorectal cancer reduction, is highlighted. The conclusions from this review are that calcium has multifaceted mechanisms of anti-carcinogenesis, but other nutrients, such as conjugated linoleic acid (CLA), lactoferrin, and folate in the dairy matrix, could also contribute. In lactase non-persistent (LNP) populations adapted to dairy foods, a bifidogenic bloom in the microbiome may add additional anti-neoplastic effects and /or increase dairy food consumption. We argue that predictions of colon cancer effects from dairy foods may be inaccurate, and that evaluating both populations together may confound outcomes.

## Introduction

Colorectal cancer (CRC) is the third and the most common cancer in men and women, respectively (1). It is also the second leading cause of death from cancer in the USA (2). Furthermore, the incidence of distal colon cancer is rising among people under the age of 55 (2). While multiple causes are involved in carcinogenesis (3–5), diet has repeatedly emerged as one possible modifiable risk factor (6). A Western diet, in particular, which is high in fats, sugar, processed foods, and low in fiber, has been identified as a contributor to many non-communicable diseases (7). Western diets have been linked to measurable inflammatory markers, which are thought to contribute to alterations in the microbiome and intestinal protective mechanisms (8, 9). As such, Western diets have high Dietary Inflammatory Indices (DIIs) (10, 11). It has been reported that CRC is related to foods with a high DII (12).

Among the different food groups, milk and dairy foods (DFs) have been the most studied in relation to CRC risk. While the role of specific DFs in reducing risks for CRC has been somewhat controversial (13–15), overall DFs are anti-inflammatory with low DII (16, 17). In fact, the majority of the findings generally support a modest protective effect of DFs against CRC (18).

This narrative review questions the dominant view of calcium as the sole effector of DF protection in all patients. A potential consequence of this concept is that other possible mechanisms are ignored. For example, in adult populations where lactase deficiency occurs, adaptation may lead to a compensatory digestion via microbial alterations. These alterations expand *Bifidobacterial* and several other beneficial bacteria. Failure to account for colonic adaptation in lactase non-persistent (LNP) populations could account for variability in outcomes (regarding CRC protection), particularly when dichotomous populations (regarding lactose digestion) are evaluated in combination. We suggest that Mendelian randomization (MR) studies evaluating the effects of DFs on CRC outcome without considering adaptation may give erroneous results toward CRC risk reduction.

We approach the question of DF reducing CRC risk from three perspectives. First, we summarize recent studies and meta-analyses examining the effects of calcium/vitamin D, milk, and dairy foods on the impact of CRC. This is to demonstrate some of the inconsistent findings in the literature. Then we review current nutrients in DFs that putatively play a role in CRC prevention. This suggests that other nutrients in DFs may contribute to CRC protection. In the last section, we review the genetics of lactase and the effects of DF consumption in lactose-maldigesting people. We discuss the impact of regular DF-containing lactose on the intestinal microflora and how this microbial adaptation could potentiate anti-carcinogenesis in individuals who have lost the intestinal ability to digest it.

## Methods

For each section of this review, we searched for English articles on PubMed and Google Scholar between the years 1980 and the present (2025). In addition, individual articles were selected from reviews based on their relevance to the topic. In the first section, reviews, systematic reviews, and meta-analyses dealing with the relationships between CRC and calcium/vitamin D, milk, dairy products, and dairy foods (DFs) such as cheese and yogurt were retrieved from the literature. In the second section, reviews and individual studies on the mechanisms by which nutrients in DFs interact with intestinal cells and the microbiome were sought. This included calcium/vitamin D effects, extracellular calcium sensing receptor (CaSR), peroxisome proliferator-activated receptors (PPARs), conjugated linoleic acid (CLA), lactoferrin, and folate. In the third section, reviews on the genetics of lactase, the differential effects of lactose in lactose-digesting and lactose-maldigesting persons, adaptation to lactose, and literature that evaluated a differential effect of DFs between lactose-digesters and lactose-maldigesters were reviewed. These included studies using Mendelian randomization. Finally, we reviewed the possible impact of lactic acid bacteria on intestinal neoplasm formation.

### Inconsistent findings on the effects of dairy foods and colorectal cancer

This section summarizes ongoing research on the effects of DFs on CRC. Garland and Garland hypothesized that vitamin D could reduce the risks for CRC (19). Newmark suggested that a high calcium intake may reduce the risk of polyps and CRC by binding bile acids (BAs) (20). This discovery led to multiple studies on the effects of calcium and vitamin D in parallel with studies on DFs, the most common foods with high calcium content. Here, we review recent studies on the use of calcium, vitamin D, and DFs to reduce the risk of colorectal cancer. It is noted that many countries add vitamin D to DFs.

#### Meta-analyses of calcium or calcium and vitamin D against CRC or polyps

Earlier epidemiological studies reported an inverse correlation between serum vitamin D levels and CRC (21–23), with some older meta-analyses quoting a 30–40% risk reduction for the highest serum levels (24, 25). Conversely, the Women’s Health Initiative showed no reduction in CRC rates in post-menopausal women randomized to calcium and vitamin D supplementation (26), nor did the VITamin D (27) and omegA-3 triaL (VITAL), which used higher doses of vitamin D (2,000 IU daily) (28). In this latter study, several cancers were evaluated, including CRC, but none of the cancers evaluated were reduced by the intervention.

In a systematic review of nine randomized-controlled trials (RCTs), evaluating the incidence of CRC in patients taking vitamin D supplementation vs. placebo, non-significant correlations were observed, not only for CRC incidence, but also for incidence of colorectal adenoma (29). A systematic review of 31 Mendelian randomization (MR) studies also showed no statistically significant association between genetically predicted vitamin D level and lower risk of CRC (30). Conversely, a meta-analysis of 28 studies evaluating the association between CRC and circulating vitamin D levels showed a 39% lowered risk of CRC for the highest compared to the lowest levels in the case–control studies vs. a 20% reduced incidence of CRC in prospective cohort studies (31). Another meta-analysis of 21 studies, covering 904,152 people, showed a lower incidence of CRC in vitamin D users [OR = 0.87 (0.82–0.92)], and improved long-term survival in the subset of patients with CRC [HR = 0.91 (0.83–0.98)] (32). In a review of 37 studies analyzing the impact of calcium and vitamin D on CRC incidence, there appeared to be a 6% decrease in CRC risk for every 300 mg of ingested calcium, and 45% decrease per 100 IU of ingested vitamin D daily (33). Importantly, a meta-analysis noted that total ingested calcium should be at least 1 g/day, and that consumption for a minimum of 10 years produces optimal results (34).

#### Meta-analyses of DFs and CRC

Yogurt has long been considered protective against CRC. A review of 45 meta-analyses investigating various dietary factors and CRC incidence demonstrated an inverse relationship of higher vs. lower intake of fiber, calcium, and yogurt on CRC risk (35). Another meta-analysis of 16 studies also demonstrated a significantly lower risk of CRC in yogurt consumers, particularly in the case–control studies (OR = 0.75, 95% CI: 0.65–0.85) (36). One hypothesis is that the *Bifidobacterium* present in yogurt has tumor suppressive effects (see below). In fact, a recent study pooling the data of two ongoing prospective cohort studies, the Nurse’s Health Study (NHS) and the Health Professionals Follow-up Study (HPFS), failed to demonstrate a statistically significant association between yogurt consumption and CRC risk, except for a trend towards lower incidence of *Bifidobacterium*-positive proximally located CRC in individuals who ate ≥2 servings/week vs. <1 serving per month (37). Still another meta-analysis of 17 (10 prospective cohort and 7 case –control) studies found that yogurt in cohort studies lowered the risk of rectal cancer (OR = 0.75, 95% CI: 0.65–0.88). In case–control studies, cheese was associated with lower colon cancer (OR = 0.89, 95% CI: 0.79–1.00) (38).

In a prospective study of 542,778 women in the UK, calcium intake was independently associated with lower CRC risk [relative risk (RR) = 0.83, 95% CI: 0.77–0.89 per 300 mg/day], while dairy milk intake was not (39). However, an earlier meta-analysis of 19 cohort studies demonstrated a non-linear dose-related significant reduction in CRC risk with total dairy product consumption, and milk intake specifically (RR: 0.83, 95% CI: 0.74–0.93), but not with cheese, possibly due to its higher fat content, which might increase bile acid levels in the colon (40).

While not an exhaustive review, these examples do show inconsistencies, keeping in mind that not all studies separated supplements from dietary intake. The inconsistencies may be explained by a variety of confounding factors, including study methods [cohort, case –control, variations in initial vitamin D levels or calcium intakes, variation in the number of different dairy products, different ways of evaluating DFs (e.g., total vs. parts of DFs)]. Also, to date, few studies have considered differential metabolism in the genetic handling of lactose.

### Theoretical elements and mechanisms in dairy foods impacting CRC

This section reviews different nutrients in DFs and postulated mechanisms of action. Table 1 outlines these different nutrients.

**Table 1:** Nutrients in milk and dairy foods with anti-inflammatory and anti-neoplastic effects that are theoretically considered to play a role in anti-carcinogenesis.

Nutrient	Effectors	References
Calcium	Binds secondary bile acids	(20, 41, 42)
	Effect on CaSR	(44, 45)
	Prebiotic effect on *Lactobacilli*	(52, 53)
	Indirect effect on PPARγ	(61)
Vitamin D	Combined effects with calcium	(47, 51)
Conjugated linoleic acid	Stimulates PPAR*γ*	(55, 56, 60, 62)
Lactoferrin	Different with iron loading	(66, 67, 69)
	Anti-inflammatory anti-neoplastic effects	(66, 67, 69)
Folic acid	Needed for normal DNA methylation	(71–73)
Mammalian oligosaccharides	Important for neonatal bifidogenic effects	(100)
Lactose	Enhances consumed quantity of Dairy foods	(91)
	Adapted LNP persons increased *Bifidobacteria* in the microbiome	(83)

CaSR, calcium sensor receptors; PPARγ, peroxisome proliferator-activated receptor γ.

#### How do calcium and vitamin D exert anti-CRC effects?

Calcium is a major nutrient that is suggested to be responsible for anti-neoplastic effects.

Newmark et al. suggested that calcium forms soaps in the intestine, which bind bile acids (BA) with fatty acids (20). The BAs are thought to induce carcinogenesis via deconjugation and dehydroxylation of primary BAs in the large bowel by anaerobic bacteria (20). The secondary BAs (e.g., deoxycholic acid) were also found to be toxic to cell systems and promote colorectal carcinogenesis (41). Dietary calcium was shown to precipitate and inactivate BA. Studies showed that the majority (95%) of the dietary calcium given to healthy participants was recovered in stool. Additionally, calcium increased bile and phosphate excretion (42). Similar findings were reported with calcium supplements (43). Precipitation of calcium salts is thought to reduce the hyper-proliferative effects of dihydroxy bile salts.

Additionally, calcium activates cellular signaling. The extracellular calcium-sensing receptor (CaSR), a G-coupled protein in the plasma membranes, was originally found in the parathyroid glands and kidneys (44). It is crucial for maintaining calcium phosphate homeostasis. Later, it was recognized to have wider, pleiotropic functions and to be present in many other cells (macrophages, eosinophils, and monocytes) as well as in other organs. In fact, CaSR is present in the lungs, breasts, prostate, and intestine/colon. This receptor controls inflammation and neoplastic transformation (in addition to its calcium/bone effects). In the lungs, it increases inflammation, while in the breast and prostate, it is considered an oncogene. Although it may also increase gastric carcinogenesis in the rest of the intestine and colon, it has opposite effects and inhibits neoplastic transformation in response to high calcium. CaSR also responds to several other stimulants, like zinc and polyamines (45). As a result, it is thought to impact on both inflammation and carcinogenesis (46). The concentration of CaSR is most plentiful in mature apical colonocytes and less in colonic pits (45). The presence of high calcium stimulates CaSR, and this is augmented by the presence of vitamin D (47).

Levels of CaSR are found to be diminished in inflammatory intestinal diseases, including experimental colitis and CRC (45, 48). In the case of CRC, stimulation of the receptor increases the transcription and secretion of the non-canonical Wnt5a, a member of the Wnt family of signaling glycoprotein molecules with multiple developmental functions (49). Wnt5a inhibits *β*-catenin reporter, which in turn decreases the canonical secretion of aberrant Wnt proteins and reduces DNA replication and carcinogen formation when APC mutations are present (46, 50).

Calcium and vitamin D, through CaSR, also potentiate claudin-2, which strengthens barrier function at apical “kissing” points of cells and thereby reduces a pro-inflammatory reaction (46, 51).

The third manner by which calcium potentiates its anti-neoplastic effects is through changes in the microbiome. *In vivo* studies have shown that calcium phosphate precipitates the cytotoxic effects of luminal contents and alters the colonic microflora, particularly *Lactobacilli*. This, in turn, was protective against pathogens (e.g., Salmonella) (52). Similar findings of induction of *Lactobacilli* were reported in a review of how dietary calcium may protect against obesity and diabetes, which are associated with increased risks for CRC (53). In addition, recent studies have also noted the importance of extracellular calcium and calcium signaling in the formation of biofilms, which protect against invading pathogens (54). A fourth manner by which calcium exerts anti-inflammatory and anti-carcinogen formation is via indirect signals that stimulate PPARγ. This is further discussed in the next section.

#### The effects of conjugated linoleic acid

Dairy foods contain several other nutrients that could impact both anti-carcinogenesis and inflammation. One of these is conjugated linoleic acid (CLA), which is a polyunsaturated fatty acid found in meats from ruminants and DFs. These fatty acids have been studied for anti-carcinogenic effects *in vitro*, in animal studies, and clinical reports, and putatively active via the peroxisomal proliferator–-activated receptor (PPAR) family of nuclear receptors (55). These ubiquitous receptors reside in the cytoplasm and, upon stimulation by a host of factors, migrate into the nucleus and combine with retinoid X by heterodimerization, leading to transcription of different genes. Three classes of receptors are recognized: PPARα, PPARβ/*δ*, and PPARγ. Variants of the PPAR family are found in multiple cells, while the PPARγ subtype primarily exerts its effects on the small bowel and colon (56).

These receptors have a wide range of pleiotropic functions connecting metabolic factors and inflammation. PPARs control insulin and glucose homeostasis. In the intestine and colon, PPARγ modulates pro-inflammatory cytokines [e.g., Tumor Necrosis Factor Alpha (TNFα), IL-6, as well as others and adhesion molecules]. PPARγ indirectly also affects DNA replication by modulating transcription as well as modifying histone deacetylation (57). The binding of PPARγ leads to cell proliferation, differentiation, and cellular homeostasis (58, 59). In several diseases, including CRC and inflammatory bowel diseases, the PPAR system is downregulated (56).

PPARγ is also related to the Wnt/*β*-Catenin system in a reciprocal fashion (60). The canonical Wnt/β-catenin system is upregulated, leading to both increased pro-inflammatory cytokine transcription and proliferation of DNA. This process is achieved by the promotion of aerobic glycolysis (Warburg effect), which is hypothesized to contribute toward neoplastic transformation (60). Stimulation of PPAR*γ* leads to inhibition of this sequence. As alluded to above, cytosolic calcium levels have an indirect impact on PPAR stimulation through stimulation of proliferator-activated receptor γ co-activator-1α via the P38/calcium/calmodulin-dependent protein kinase system in an animal model (61).

In a mouse model, PPARγ-deficient mice and mice displaying this receptor underwent controlled feeding experiments using 1-g conjugated linoleic acid (CLA)/100 g. When compared after induction of colitis, there were fewer cases of colitis and colon adenocarcinoma formation in the wild-type mice. The authors concluded that CLA likely works through PPARγ (62). However, translational relevance to humans requires further studies.

The PPARγ system also responds and is upregulated by different lipids and thereby downregulates cellular proliferation (63). Of note is that this nuclear receptor also responds to microbial production of short-chain fatty acids (SCFAs), opening the mechanism for anti-inflammatory and anti-neoplastic effects of short-chain fatty acids (SCFAs) produced by many bacteria (62). In addition, *Bifidobacteria* species produce CLA (64). In humans, the role of PPARγ in reducing neoplastic effects is inconsistent, with no evidence that therapy targeting this receptor once disease is established is more consistent and would not be useful by itself as therapy (65). It may be, however, better in prevention.

#### Other nutrients with anti-neoplastic effects in dairy foods

Other nutrients in milk with a potential for anti- inflammatory and anti-neoplastic properties are the multifunctional iron-binding glycoprotein found in mammalian milks (66). Bovine lactoferrin has similar properties to human lactoferrin, which has been used in therapeutic roles in some inflammatory conditions (67). However, the content in bovine milk is much less compared with human milk (66). Lactoferrin has different functions on bacteria depending on whether it is in the free form or bound to Fe^3+^. While it may provide iron for bacterial growth in the iron-chelated state, it inhibits growth in the iron-free state. By binding LPS, this prevents activation of inflammatory cells. In addition, lactoferrin can bind nuclear factor kappa-B (NFκB), and as such, it has both immunomodulatory and anti-neoplastic functions (68).

Another example where lactoferrin downregulates humoral and cellular immunity is in a mouse model of dextran sodium sulfate (DSS)-induced colitis, which again may be different from human experience. Lactoferrin decreased IL-1β and TNFα (69). In the Adenomatous Polyposis Coli ‘Floxed’ (APCF) mouse model of DSS-induced colitis and dysplasia, lactoferrin inhibited neoplastic transformation. Lactoferrin has also been used to decrease cellular proliferation, induce apoptotic cytokine responses [interleukin (IL-18), caspase-1, caspase-3, and caspase-8], to induce natural killer cells, and to induce anti-angiogenesis (70).

In addition to the above-mentioned nutrients, small amounts of folic acid are also present in DFs, particularly fermented products (71). Folic acid has effects on normal DNA formation and epigenetic function (72, 73).

While different nutrients in DFs are listed, the actual mechanism of protection is postulated to be predominantly calcium (and vitamin D). This is based on the results of clinical studies evaluating the impact on CRC. Calcium alters the carcinogenic effects of bile salts and interferes with cell signaling; as such, it has a dual effect against CRC. Nevertheless, it is difficult to rule out the possibility that the dairy matrix, which includes all the different elements detailed here. It is equally difficult to evaluate the magnitude of clinical contribution of each of the other nutrients.

### Effects of dairy foods in lactose-maldigesting populations

There is a general conception that adults who are genetically programmed to have reduced lactose digestion ability consume less milk and DFs than those who retain the ability to digest it. As a result, the potential protective effects of DFs are putatively reduced in these populations. However, as will be discussed here, there are mitigating factors that allow maldigesters to consume more DFs via a route affecting their microbiome. On a functional basis, the world is divided into people who retain the ability to digest the disaccharide lactose into adulthood [25–30% lactase persistent (LP)] and people who lose the ability to digest lactose by early adolescence [75–70% lactase non-persistent (LNP)]. A fundamental question is whether this dichotomy impacts the effect of DF on diseases like CRC.

The LP status is due to a dominant genetic mutation on chromosome 2q21 in the minichromosome 6 transcription region, away from the lactase phlorizin hydrolase (LPH) gene. Adults with LP mutations retain lactase activity into adulthood and have no difficulties with consuming DFs. In contrast, some LNP persons do experience largely gastrointestinal symptoms from consuming DFs randomly. There are five major geographically distributed polymorphisms responsible for this phenotypic dichotomy (14010*C, 14009*G, 13915*G, 13910*T, and 13907*G) (74). The first of these described was the 13910*T polymorphism, which is found in the majority of the Europeans and their descendants in the New World, as well as some Asian people (e.g., Japanese). There are some other polymorphisms that require further evaluation but are beyond the scope of this review.

Partly because of this phenotypic dichotomy, there is some variation in milk and DF consumption. The population frequency of LP and LNP global distributions impacted global milk and DF consumption (75, 76). In earlier studies, it was observed that there was an ecological relationship between DF consumption and CRC mortality (and, by extension, incidence) rates globally. The rates of CRC mortality and DF consumption were highest in high LP and high dairy-consuming countries (74, 75). The ecological nature of these observations is weak, but they showed geographic trends. More recently, because of large population shifts mostly from south and east to west and north, the LP, LNP populations in Western societies and North America have changed with regard to DFs consumption (77). Indeed, studies evaluating the relationship between CRC and the European genetic polymorphism TT-13910 (rs4988235) showed that the dominant TT genotype is associated with lower risks of CRC while the CC genotype is associated with increased CRC risk (77, 78). These studies did not directly consider DFs consumption. A large study by Papier et al. compared genetically estimated DFs intake with actual findings in LP and LNP. While LP reduced CRC risk by 40%, LNP reduced it by only 14% without examining DF intake. When the actual data were examined, the real reductions in risk were 17% for a daily calcium intake of 300 mg and 11% for the highest milk intake (39). It should be noted that while genetics is involved with both Ca and lactose assimilation, there is no link, other than reduced intake in LNP persons, between the two nutrients.

The ecological observations quoted above were an example, however, of an ecological fallacy, because DFs are generally reported to reduce CRC risks (75, 76). It should be noted that these studies mostly suggest that the high calcium contents of DFs consumed (over 900 mg and as much as 1.2 g) is the primary mechanism of anti-carcinogenesis (34, 39). Total calcium could be obtained from diet and/or supplements; however, the reduction in CRC risk was also observed for milk and total DF consumption (40). Mendelian randomization (MR) studies suggest that the dominant genotypes are associated with lower CRC risks (38, 77, 78), due to higher consumption of DFs. Relatively few studies have actually compared Df intake and outcome between high-frequency LP/high DF intake and high LNP/low DF intake populations. A unique older meta- analysis of 80 publications did compare DF effects on CRC outcome (highest vs. lowest intake) and suggested that the relative risks in high LP (≥80% LP) and high LNP (≥80% LNP) countries were similar (RR = 0.80, 95% CI: 0.73–0.88, and 0.84, 95% CI 0.73–0.97). Among countries where LP and LNP distributions were mixed (average LP/LNP, 50%) there was a non-significant numerically modest protective effect (RR, 0.92, 95% CI: 0.79–1.06) (79). The explanation offered for the similar reduction in risk was that LNP persons developed colonic adaptation to regular lactose ingestion (80). This may compensate for lower calcium intake in LNP. The explanation for the statistically insignificant reduction in relatively similar mixed populations was that the calcium effect could involve a threshold. In fact, this hypothesis is supported by the report of Aune et al., where a non-linear dose effect to calcium was found (40).

Colonic adaptation to lactose was shown to involve the emergence of lactic acid-producing bacteria with low hydrogen production capacity, *Lactobacilli* and *Bifidobacteria in vitro* and *in vivo* (81). In a small pilot study, consumption of an aqueous solution of lactose in LNP participants led to a significant emergence of *Bifidobacterial* species after 2 weeks (82). More recently, 3 months of lactose consumption in LNP participants showed a doubling of 22 species of *Bifidobacteria* as well as *Anaerostipes* species, confirming earlier observations (83). While *Lactobacillus* species increased as well, the numbers were much lower. Similar comparisons in LP populations given lactose did not produce any microbial response (82).

In another pilot study, breath hydrogen response to a 50-g lactose load correlated inversely with pretest DFs intake (84). In Japan, increased colony counts of *Bifidobactaria* species in colonic microflora were positively correlated with DFs consumption, suggesting that the greater DFs (with lactose) intake, the greater the population of *Bifidobacteria* (85). Table 2 outlines evidence showing the effects of (intestinal) colonic adaptation resulting in a lactic acid bloom of microflora, with regular lactose consumption in LNP persons.

**Table 2:** Evidence for a bifidogenic effect of lactose in lactase non-persistent populations.

Type of study	Outcome	References
*In vitro*	Reduced hydrogen production due to lactic acid bacteria	(80, 81)
Epidemiology	Japanese have increased *Bifidobacteria* in stool, which correlates positively With dairy food consumption, in 1,068, all LNP	(85)
	A Genetic study of almost 6,000 Europeans finds the recessive CC of the Lactase gene associated with increased colonic *Bifidobacteria* in conjunction with dairy food consumption	(86)
	Meta-analysis of 80 studies grouped into dominant frequencies of LP and LNP in populations suggest similar risk reduction by total Df	(79)
*In vivo* trials	Small 4-week feeding of 25-g lactose to 23 LP and 18 LNP significant increase in *Bifidobacteria* species in LNP Only	(82)
	Small 3-month trial of 25-g lactose in 25 Asians with LNP status doubling of 22 species of *Bifidobacteria* and *Anaerostipes*	(83)
	Specific *Bifidobacteria* (the four most common) and three *Lactobacilli* stimulated	
	*B. adolescentis*	*L. Schleiferilactobacillus*
	*B. longum*	*L. Companilactobacillus*
	*B. pseudocatenulatum*	*L. Lapidilactobacillus*
	*B. bifidum*	

We note above that the use of MR for lactase genetics suggests that CRC is more common in recessive individuals than in those with the dominant lactase genotype. However, it is emphasized that the reduction in CRC risk is wholly related to DFs consumption. Neither the dominant nor the recessive genotypes have been independently linked with CRC. A study evaluated 5,959 genotyped patients and their related bacterial microflora in relation to different genes. It showed that the European gene recessive to LCT (LNP) in conjunction with dairy intake had a strong relationship with *Bifidobacteria*. This was not the case with LP persons (86).

Here, we demonstrate some issues that confound studies related to DFs and CRC (87–89). Table 3 highlights 4 studies that are used in a hypothetical sense to crudely compare outcomes (39, 40, 90, 91). First¸ there is a cultural factor that determines the amount of milk drinking in Asian countries (which are predominantly LNP) (92). Cultural refers to learned local traditions, social norms, and historical factors rather than necessity or availability (93). A recent Chinese study suggested that DFs increased risks for CRC, although not significantly (90). The Fukuoka, Japan study evaluated both calcium and milk intake in a smaller population and found an OR of 0.62 for the highest intake of calcium and 0.6 for milk (91). While an OR may overestimate the RR, a correction is required only when the RR is 0.5 or 2.5 for the condition, which is >10% (94). In Japan, CRC rates are still less than 10% for men and women’s lifetime risk (95).

**Table 3:** Hypothetical comparisons of milk and their calcium and lactose contents [based on bovine milk] (88) among studies emanating from previously dominant LP and LNP regions.

Author	Region	Risk ratios Calcium	Risk ratios Dairy	Risk ratios Milk	Amount Milk/DF g OR Cal/day, mg	Content Calc mg	Content Lact g*
Kakkoura (90)	China	NS	HR: 1.09 (1.01, 1.18) (p 0.06)	DF mostly milk	80 50/day	100 62.5	3.6–4 2.4
Papier (39)	UK	RR: 0.83 (0.77–0.89)	NS	RR: 0.89 (0.84–0.94)	Milk, 282 Cal, 300	352 375 (50/d)	13.8–14.1 15/day
Mizoue (91)	Japan	OR: 0.62 (0.45, 0.85) p.002	NS	OR: 0.6 (0.4, 0.89) p0.46	>200 g/day	>250 mg	> 9.7–10 g
Aune (40)	Mixed Majority of the LP	NS	RR: 0.83 (0.78, 0.88)	RR: 0.91 (0.85–0.94)	400 DF 200 milk	328 250	DF not itemized 9.7–10

NS, not stated; HR, hazard ratio; OR, odds ratio; R, relative risk; Cal, calcium; Lact, lactose; DF, dairy food; LP, lactase persistence. *This note is added to clarify why lactose in LP persons is not bifidogenic based on quantities consumed. It was determined that LP persons would maldigest about 8% of the lactose consumed (4-g lactose/L of milk, which is 1,030 g). This compares to 42–75% maldigested/malabsorbed in LNP persons [21-–37.5-g lactose in 1,030-g milk (89)]. The threshold dose of lactose to begin observable adaptation is about 6 g/day (87).

The milk intake estimates in the three positive studies approximate the quantities. The amount of calcium and lactose in the study by Kakkoura et al. is minimal and may be negligible for either calcium or lactose adaptation effects (90). The study does not suggest an increased risk of CRC from DFs but may show a lack of any protection due to small quantities.

The second point is the results of the Fukuoka study, which show that milk intake can match that of LP persons. Note that the MR calculation by Papier et al. based on estimated DF intakes found a 14% benefit in LNP and 40% in LP (39). The Japanese study’s outcome exceeds that of the calculations for LNP, supporting the notion that, with adaptation, LNP can achieve average intakes approaching those of LP. In another proof of report, a LNP person of Italian descent was de-adapted from a 500-g milk/day diet. While breath tests were normal initially, after a few weeks of avoiding DFs, a breath test result showed lactose maldigestion, unmasking true genetic status (96).

The third point is that the hypothetical MR, 40% reduction of risk in LP populations, exceeds the risk reduction in Papier’s (39) study as well as in Aune’s (40), Szilagyi’s (79) and another publication (97). Given the variability in milk consumption published in the USA (77) and migrations from large LNP populations in the last three-to-four decades, it may be assumed that studies from previously estimated majority LP populations are more inhomogeneous.

Given these observations, the use of MR to assess CRC risk and the effects of DFs may be inaccurate without accounting for true DF intake.

Mendelian randomization uses genetic variants as instrumental variables to link causality to a modifiable variable, reducing confounding and reverse causality. Several assumptions must be met to use this technique. If the primary pathway that is controlled by the genetic variant can be bypassed by another pathway that leads to the same outcome, so-called horizontal pleiotropy, then the MR is invalid (98). In the case of lactase, if lactose can be digested independently of LPH, as with or by bacterial galactosidase, it qualifies as horizontal pleiotropy, which negates MR’s findings.

### Theoretical effects of probiotic-type bacteria against colorectal cancer

In this section, mechanisms by which specific species of bacteria induced by adaptation exert anti-neoplastic effects are summarized. It is emphasized that a large majority of these studies are conducted *in vitro* and many in animal models, and that, as such, relevance to human translation requires further evaluation.

As noted, long-term DF consumption (containing normal amounts of lactose) in LNP persons leads to an increase mainly in populations of lactic acid-producing bacteria. This does not seem to occur in LP populations. The main bacteria stimulated are from the phylum Actinobacteria, specifically the genus *Bifidobacterium*, with multiple species (at least 22 known). To a much lesser extent, phyla Firmicutes, genus *Lactobacillus*, and several species (at least 3) of this group are also stimulated (83).

Species of *Bifidobacteria* dominate the microflora of breast-fed neonates, and this genus is associated with protection against some diseases, both at the neonatal level and for some diseases in later life (99, 100). In breastfed infants, the microflora is controlled by a host of mammalian oligosaccharides that escape digestion and absorption in the neonate (101).

*Bifidobacteria* have been extensively studied *in vitro* and *in vivo* for probiotic effects against numerous diseases, including CRC (102). *Bifidobacteria* have been shown to theoretically prevent colorectal cancer through five principal mechanisms. First, *Bifidobacteria* modulate intestinal microbiota. Second, they appear to promote apoptosis of cancer cells. Third, they downregulate oncogenes. Fourth, *Bifidobacteria* regulate the immune system. Finally, *Bifidobacteria*, along with other bacteria, help maintain normal bile acid circulation.

*Bifidobacteria* modulate intestinal microbiota by competing with pro-carcinogenic organisms. Shang et al. collected fecal samples from AOM/DSS CRC mouse models with and without exposure to *Bifidobacterium longum*. The presence of other bacterial species was altered, with significantly fewer *Alistipes* (pro-carcinogenic) and significantly more *Lachnospiraceae* (anti-carcinogenic) in the CRC mice exposed to *B. longum* (103). They further found that bacterial operational taxonomic units (OTUs) varied significantly between the two groups, suggesting that the overall gut microbiome was significantly different due to the administration of *B. longum* (103).

*Fusobacterium nucleatum* and its metabolites are associated with colorectal cancer development. Xu et al. demonstrated that the metabolites of six species of *Bifidobacteria*’s cell-free supernatant (CFS) can inhibit the growth and membrane formation of *F. nucleatum* (104). However, in CRC cells infected with extracellular vesicles derived from *F. nucleatum* (FnEVs), [a promoter of colorectal cancer, proliferation, and oxidative stress], CFS inhibited some oncogenes while activating others. This suggests that while it has positive effects on the gut microbiome, effects on the oncogenic pathways investigated may be milder in the context of CRC cells already infected with FnEVs (104).

Apoptosis is a key physiological anti-tumor process. Evidence suggests that Bifidobacteria have a pro-apoptotic effect on colorectal cancer cells. An *in vitro* study using human colon cancer cell lines, Faghfoori et al. evaluated cancer cell survivability in the presence of metabolites from several *Bifidobacteria* species (105). This group also investigated the expression of several known pro-apoptotic and anti-apoptotic genes in the presence of these metabolites. Their results indicated that metabolites from *Bifidobacteria* species, including *Bifidobacterium adolescentis*, *Bifidobacterium animalis* subsp*. lactis*, *B. animalis* subsp*. animalis*, *Bifidobacterium bifidum*, and *Bifidobacterium angulatum* promote colorectal cancer cell apoptosis. *B. bifidum* had the strongest effect, inducing apoptosis in 79.78% of Caco-2 cells 53.32% of HT-29 cells.

Metabolites also were shown to downregulate anti-apoptotic and upregulate pro-apoptotic genes (105). Soraya et al. investigated the pro-apoptotic effects of *Bifidobacterium* cell extracts on colon cancer cells *in vitro*, with an additional focus on apoptotic signaling pathway activity (106). *Bifidobacteria* were shown to have cytotoxic effects on CRC cells, with cell-free supernatants having higher anti-cancer effects than bacterial cell extract, implying that bacterial secretions are responsible for the proapoptotic effect of *Bifidobacteria*, as opposed to cellular components (106). Researchers also noted significantly higher levels of Caspase-1, Caspase-3, and Caspase-9 in the presence of certain *B. bifidum* cell-free supernatants. Caspase-3 and Caspase-9 play significant roles in the mitochondrial pathway of apoptosis, while Caspase-1 is traditionally associated with pyroptosis (inflammatory programmed cell death by ruptured cell membrane) (107).

Further evidence supporting Caspase-mediated apoptosis of CRC cells in the presence of *Bifidobacteria* comes from an animal study by Shang et al., which demonstrated that the combination of *Bifidobacterium* H3-R2 and *Lactococcus lactis* KLDS4.0325 caused apoptosis via increased levels of Caspase-3 and Caspase-9 (108). The combination of these 2 strains led to increased effect as compared to each strain alone, suggesting that *B. Bacterium*’s pro-apoptotic effects may be enhanced when in synergy with other protective microorganisms (108).

There is evidence to suggest that *Bifidobacteria* downregulate CRC oncogenes. An *in vitro* study investigated the impact of *Bifidobacterium* cell-free supernatant on gene expression of colon adenocarcinoma (Caco-2) cells. Results indicated that expression of *β*-Catenin, PI3K, TGF-*α*, TGF-β, Bcl2, TLR4, and CEA was significantly reduced in the presence of cell-free supernatant (109).

*Bifidobacteria* have been shown to directly inhibit oncogenic signaling pathways. Parisa et al., in a mouse study, demonstrated that a five-species cocktail of *Bifidobacteria* significantly downregulated epidermal growth factor receptor (EGFR), Human Epidermal Growth Factor Receptor 2 (HER-2), and cyclooxygenase-2 (COX-2), key oncogenic drivers of CRC (110).

The exact bacterial metabolites that may alter the expression of these genes have not yet been elucidated. Another possible mechanism is via regulation of the mitogen-activated protein kinase kinase/extracellular signal-regulated kinase (MEK/ERK) cascade, a known oncogenic cascade. An animal study investigated the effect of *Bifidobacteria* on the MEK/ERK cascade in gliomas through transcription sequencing, demonstrating that this pathway is inhibited in the presence of *Bifidobacteria* (111). Although the effect on this pathway was investigated in glioma, not CRC, MEK/ERK is typically hyperactivated in CRC (112).

*Bifidobacteria* play an essential role in the production of butyrate through the production of acetate and lactate, which is further metabolized (by a process of cross-feeding) into butyrate by other bacteria (113, 114). A review article summarized the effects of butyrate on oncogenic signaling pathways (115). Butyrate has been shown to have anti-cancer effects in both *in vitro* and animal studies (115–117). For example, butyrate is a histone deacetylase (HDAC) inhibitor. These key proteins determine which genes are transcribed by deacetylation of histones (118). When regulatory genes are acetylated, they undergo transcription. Therefore, by inhibiting HDAC, butyrate promotes the transcription of regulatory genes that can regulate proto-oncogenes. Importantly, HDAC8, also inhibited by butyrate, is a key promoter of the JAK2/STAT pathway, which promotes cellular proliferation (119). Additionally, butyrate has been shown to hyperactivate the Wnt signaling pathway, which regulates levels of *β*-catenin, and could initiate colorectal cancer when it is dysregulated. When hyperactivated by butyrate, this pathway instead results in CRC cell apoptosis (120). Various other pathways are described by Chen et al., who further suggest that, by inhibiting multiple interacting pathways, butyrate exerts an overall inhibitory effect on tumorigenesis (115).

In addition, as stated, species of *Bifidobacteria* can produce conjugated linoleic acid (64). Both short-chain fatty acids and CLA up-regulate PPARγ (63, 64).

*Bifidobacteria* regulate the immune system. A clinical trial in CRC patients demonstrated that probiotics, including *Bifidobacterial* species, reduced systemic levels of proinflammatory cytokines TNF-*α*, IL-6, IL-10, IL-12, IL-17A, IL-17C, and IL-22 (121). In addition, a mouse model study found that *Bifidobacteria* significantly upregulated 236 and downregulated 195 messenger RNAs (mRNAs), including those related to several immune and tumor pathways (103). Various mechanisms have been proposed. *Bifidobacterium* and *Lactobacilli* have been shown to activate the NF-kB signaling cascade, which promotes immune system balance by activating pro-inflammatory and anti-inflammatory cytokines, thereby permitting appropriate immune responses while preventing excessive activation (122, 123). Furthermore, butyrate, promoted by *Bifidobacteria*, inhibits CD8 + activation by suppressing IL-12 production by dendritic cells (124). Additionally, a recent study suggests that *Bifidobacterium infantis* supernatant upregulates PD-L1 and thereby downregulates the PI3K-Ack-mammalian target of Rapamycin (mTOR) pathway, ultimately creating an immunosuppressive effect (125).

*In vitro*, several studies were published showing anti-cancer effects of *Lactobacilli* (126–128), including a study suggesting a cocktail effect similar to *Bifidobacteria* (129). The microbiome, including both *Bifidobacteria* and *Lactobacilli*, also participates in reducing toxic secondary bile acids such as deoxycholic acid (130, 131).

Few human studies have been carried out on CRC for either *Bifidobacteria* (132) or *Lactobacilli* (133) and interest in this topic remains high. Table 4 outlines anti-neoplastic *Bifidobacterial* effects.

**Table 4:** Studies with *Bifidobacteria* showing anti-inflammatory and anti-carcinogenesis mechanisms.

Mechanism	Specific effects	Reference
Modulation of intestinal microbiota	Reduced *Alistepes*	(103)
	Metabolite reduces *F. nucleatum*	(104)
Promote apoptosis of cancer cells *in vitro*	Metabolites from *B. adolescentis*	(105)
	*B. animalis*, *B. bifidum*, *B. angulatum*	(106)
	Increases Caspase-1, −3, and −9	(107)
*B. bifidum* and lactis *In vivo*	Cell-free supernatant Increases Caspase-3 and −9	(103, 104)
Downregulate oncogenes	Cell-free supernatant in Caco-2 cells reduces β-Catenin, PI3K, TGF-*α*, TGF-β, Bcl2, TLR4, and CEA	(109)
	In mouse, downregulation of oncogenes EGFR, HER-2, and COX-2,	(110)
	*In vivo* MEK/ERK cascade is inhibited	(111)
	Production of butyrate via cross-feeding interferes with oncogenic signaling	(113–117)
	Including JAK-2/STAT promoter of cellular proliferation	(119)
	Hyperactivation of the Wnt/β-catenin system Results in cancer cell apoptosis	(115, 120)
	*Bfidobacteria* produce CLA and Butyric acid Both upregulate PPARγ	(63, 64)
Regulate the immune system	In a clinical study *Bifidobacteria* Reduced levels of TNF-α, IL-6, IL-10, IL-12, IL-17A, IL-17C, and IL-22	(121)
	Species of *Bifidobacteria* and *Lactobacilli* Activate NFκB, keeping a pro and anti-inflammatory balance	(122, 123)
	*Bifidobacteria* through inhibition of IL-12 and downregulation of PI3K-Ack-mTOR pathway, creates immunosuppressive effects	(124, 125)
Maintain normal bile acid circulation	Both *Bifidobacteria* and *Lactobacilli* reduce toxic effects of deoxycholic acid	(130, 131)

The large majority of these pathways were shown in *in vitro* studies or animal models, except for reference (120).

Important observations from studies on lactic acid bacteria include effects that are likely more evident in “cocktail” combinations. The recent confirmation that multiple species of *Bifidobacteria* and other bacteria are stimulated by lactose (83) lends these organisms a potentially significant contribution to disease modification.

## Discussion

This review summarizes recent publications on the effects of calcium, vitamin D, milk, and DFs on the prevention of CRC. While there is still debate on the effects of specific DFs (e.g., cheeses and yogurt) and specific sites affected (rectal, colon, and distal proximal sites), studies do support that the majority of dairy likely has some risk-reducing effects (35, 37) albeit with a low grade of confidence (134).

Among studies, there appears to be agreement that high calcium, with or without added vitamin D, is the likely the mediator of anti-CRC effects. Overall, these protective levels can be achieved with diets high in calcium (39) or with supplements (33), which need to be continued for about 10 years before the outcome is realized (33). It should be noted that calcium also has multiple impacts, both on reducing initiation of carcinogenesis (precipitation of BAs) and on cellular responses to initiators (CaSR anti-neoplastic and anti-inflammatory), in addition to those mentioned above.

Much of the cellular information has not been validated in the human condition, however.

We review various nutrients in DFs that may also contribute to anti-carcinogenesis and the putative mechanisms. As such, we suggest that perhaps the matrix hypothesis of the DF effect might be a more complete explanation for anti-neoplastic effects (135). Further work will be needed to tease out whether this approach is valid.

An important element discussed here is the possible effect of adult-onset genetic lactose maldigestion on CRC. As stated, regular lactose consumption in LNP persons could alter the microbiome toward a more favorable bifidogenic population. The demonstration that *Bifidobacteria*, across multiple *in vitro* and animal studies, could lay the foundation to evaluating these *in vivo* in humans to determine whether this change favors anti-neoplastic effects.

Failure to take this factor into account may introduce a confounder when analyzing the effects of DFs on CRC (and likely other diseases affected by microbiome contributions). The consequence of this, besides a possible anti-carcinogenic effect, is to increase the amount of DFs by LNP persons, therefore approaching levels of intake by LP persons (84).

As we discussed above, it is not yet clear whether calcium has a threshold effect. At this time, it is not established what level of colonic adaptation over what period begins the reduction in CRC risk. In the earliest study examining this dichotomous response suggested that the larger the contribution from each LP or LNP group analyzed together, the greater the dilution of the reduction of risk (38). The most recent analysis by Papier et al. also found a discrepancy between the predicted reductions of risk in LP and LNP, and what was actually found (136). This possible dilution effect should be further evaluated in the future.

However, because the microbial metabolism of maldigested lactose offers an alternate pathway for the lack of intestinal lactase, the use of the Mendelian randomization method to predict response to dairy foods is probably invalid. As a result, analyzing LP and LNP populations together is likely to yield inaccurate outcomes.

### Additional knowledge gaps

**Figure 1:**
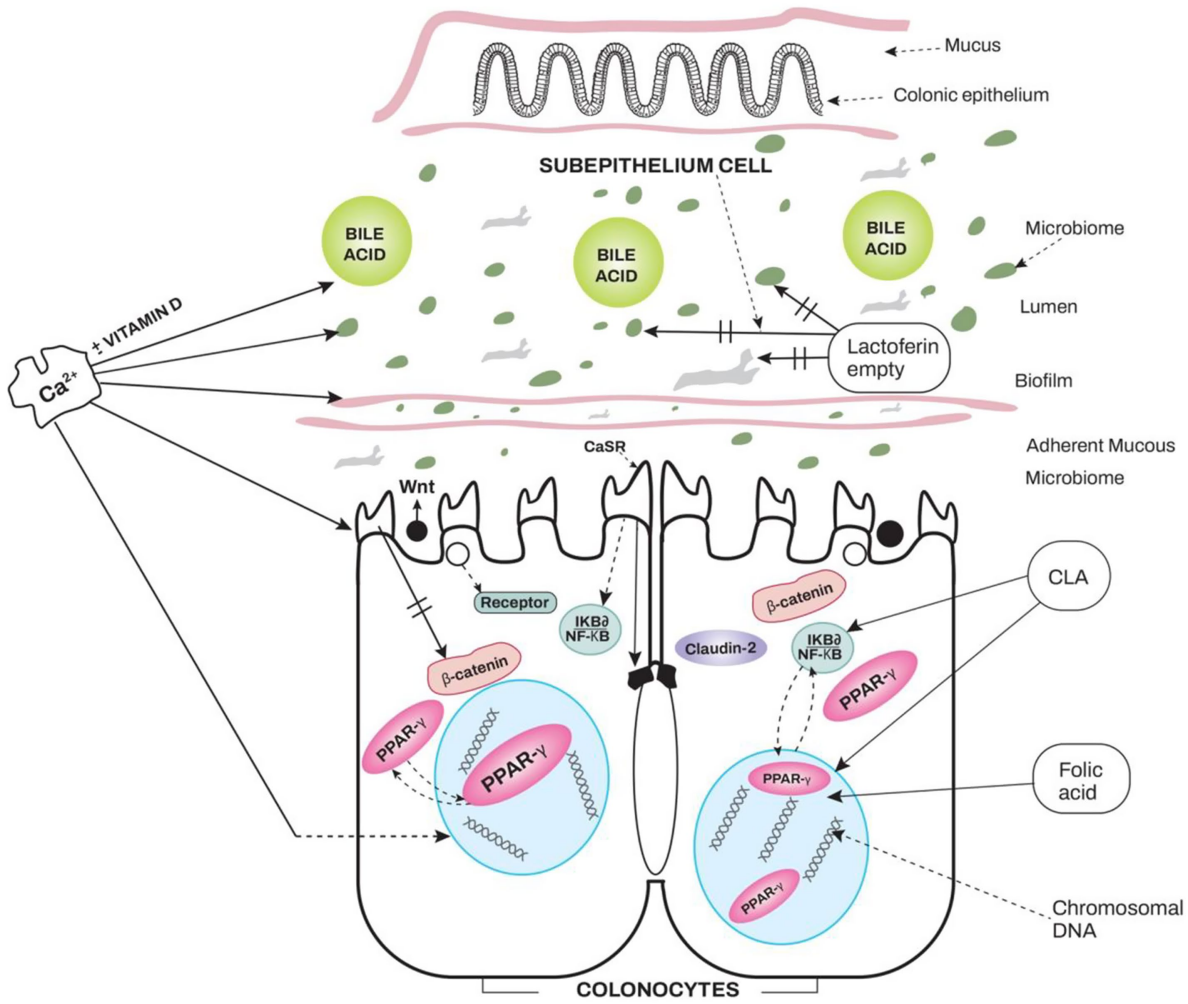
Interaction of four main nutrients in milk and dairy foods with luminal bacteria, other molecules and cellular receptors are shown. The four nutrients are Calcium (Ca++ with or without intervention of vitamin D), lactoferrin (without binding to ferric iron molecules), conjugated linoleic acid (CLA) and folic acid. These features are demonstrated in two apical colonocytes which display extra cellular calcium sensing receptors (CaSR), cell surface attached Wnt protein connected to Wnt (Frazzle) receptors, cytoplasmic β-catenin dependent canonical Wnt system. Proliferator Activated Receptor gamma (PPARϒ), which is present both in the cytoplasm and nucleus, is also displayed. The nuclear factor kappa-B (NFκB) molecule combined with inhibitor of nuclear factor kappa-B alpha (IKBα) is also shown in the cytoplasm. When this protein is degraded, NFκB is trans located to the nucleus, where it activates genes involved in the formation of inflammatory cytokines. Calcium forms soaps with bile acids, reducing a hyperproliferative effect of secondary bile salts on DNA (42, 43). Calcium up regulates the extracellular calcium receptor (CaSR) which in turn stimulates the non-canonical Wnt5a molecule (45, 46). This effect on the Wnt system is potentiated by vitamin D (46). In turn the stimulated Wnt5a leads to the inhibition of the canonical Wnt/β-catenin pathway and reduces DNA replication and transcription. The interference with β-catenin also down regulates the inflammatory responses. CaSR, indirectly up regulates PPARϒ (48, 50). PPARϒ also regulates inflammatory responses (56). Through the CaSR system, calcium contributes to Claudin -2, an integral component of tight junctions (45). Calcium also promotes the growth of Lactobacilli, Bifidobacteria and the biofilm in the microbiome (52–54). Lactoferrin can inhibit bacterial growth in its empty form. It can bind lipopolysaccharides (present in walls of gram-negative bacteria) and prevent its translocation. It also downregulates immune reactions (66, 68). Conjugated Linoleic acid (CLA) interacts with NFκB and PPARϒ to downregulate inflammatory responses as well as to regulate the Wnt/ β-catenin system in a reciprocal fashion (56, 60). Folic acid regulates DNA via direct production of nucleic acids and epigenetic control of DNA (71, 72). Arrows with bars show inhibition, those without bars show stimulation, and broken arrows show translocations. Location of several receptors on colonocytes and other cells was based on questions asked of Open Evidence and Google Gemini AI assistant (65, 93).

Additional research would help define the role of this selective prebiotic effect of lactose in LNP populations. A relevant question is whether the effect of lactose is generalizable to all LNP populations. Are microbial changes similar in different geographic regions? Another relevant question is whether the magnitude of the effect of this altered flora can impact colorectal cancer and other disease states where dysbiosis is prevalent. Hypothetically. long-term studies comparing lactose consumption with a probiotic-like lactose effect for polyp or CRC outcomes would be ideal but difficult. In addition (to pre and probiotic research), further studying the microbiome of LNP lactose-adapted persons compared with LNP non-adapted and LP subjects for carcinogenic potential may help focus microbial benefits or nil effects in such LNP-adapted persons (Figure 1).

## Conclusion

This review supports the beneficial effects of dairy consumption, modestly reducing the risk of CRC. While calcium affects several pathways in countering carcinogenesis, at least other nutrients in DFs could also theoretically contribute, suggesting a matrix effect of DFs.

In the case of LNP populations, the protective role of a microbial bifidogenic remains poorly defined. The contribution to anti-neoplastic effects of adaptation is a possibility that needs to be further explored in human studies. At the very least, microbial adaptation enhances the quantities of milk and DF consumption. As a result, evaluating mixed LP and LNP populations together for any protective effect of DFs on CRC and possibly other diseases related to the microbiome may dilute the statistical outcome.
